# Exploring the depth and breadth of the genomics toolbox during the COVID-19 pandemic: insights from Aotearoa New Zealand

**DOI:** 10.1186/s12916-023-02909-4

**Published:** 2023-06-14

**Authors:** Michael Bunce, Jemma L. Geoghegan, David Winter, Joep de Ligt, Siouxsie Wiles

**Affiliations:** 1 Institute of Environmental Science and Research, Kenepuru, Porirua, 5022 New Zealand; 2 Department of Conservation, Wellington, 6011 New Zealand; 3 Department of Microbiology and Immunology, University of Otago, Dunedin, New Zealand; 4 Bioluminescent Superbugs Lab, Department of Molecular Medicine and Pathology, University of Auckland, Auckland, New Zealand; 5 Te Pūnaha Matatini, Auckland, New Zealand

**Keywords:** Genomics, COVID-19, SARS-CoV-2, Genomic surveillance, Whole genome sequencing, Wastewater surveillance, Variants

## Abstract

**Background:**

Genomic technologies have become routine in the surveillance and monitoring of the coronavirus disease 2019 (COVID-19) pandemic, as evidenced by the millions of SARS-CoV-2 sequences uploaded to international databases. Yet the ways in which these technologies have been applied to manage the pandemic are varied.

**Main text:**

Aotearoa New Zealand was one of a small number of countries to adopt an elimination strategy for COVID-19, establishing a managed isolation and quarantine system for all international arrivals. To aid our response, we rapidly set up and scaled our use of genomic technologies to help identify community cases of COVID-19, to understand how they had arisen, and to determine the appropriate action to maintain elimination. Once New Zealand pivoted from elimination to suppression in late 2021, our genomic response changed to focusing on identifying new variants arriving at the border, tracking their incidence around the country, and examining any links between specific variants and increased disease severity. Wastewater detection, quantitation and variant detection were also phased into the response. Here, we explore New Zealand’s genomic journey through the pandemic and provide a high-level overview of the lessons learned and potential future capabilities to better prepare for future pandemics.

**Conclusions:**

Our commentary is aimed at health professionals and decision-makers who might not be familiar with genetic technologies, how they can be used, and why this is an area with great potential to assist in disease detection and tracking now and in the future.

Aotearoa New Zealand’s (population approx. 5.1 million people) first official COVID-19 case was reported on 28 February 2020 [1]. On 19 March 2020, with 28 confirmed cases, the country’s borders closed to all but citizens and permanent residents [2]. Shortly after, New Zealand began pursuing an elimination strategy for COVID-19, establishing a (hotel-based) managed isolation and quarantine (MIQ) system for all international arrivals [2]. It was at this time that rapid genomic surveillance capability for SARS-CoV-2 was established; prior to this, the country had very limited capability.

By December 2021, New Zealand had experienced just 11,992 confirmed COVID-19 cases and 44 deaths [3]. With high vaccination rates, the country moved to a suppression and mitigation strategy for COVID-19 (Fig. 1) [3, 4]. This ended in September 2022, and New Zealand’s borders reopened to travellers (Fig. 1) [4]. At the time of writing, masks are still required in some settings and people who test positive for COVID-19 are still required to isolate.

**Fig. 1:**
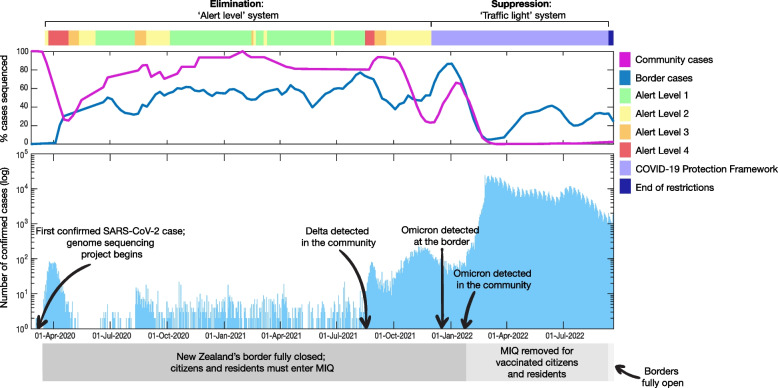
Percentage of border and community cases sequenced (top panel) and number of daily confirmed COVID-19 cases by reported date (bottom panel) in Aotearoa New Zealand. The timing of public health measures under the elimination (Alert Levels 1–4) and suppression strategies (COVID-10 Protection Framework) are also shown as are the arrival of Delta and Omicron, and national border closures

To assist its COVID-19 response, New Zealand first set up and then scaled its use of genomic technologies. New Zealand employed a variety of ‘modes’ afforded by the genetic and genomic toolkit (Fig. 2) and used this data in real-time decision-making. At this time, international arrivals coming in via MIQ were the only source of COVID-19. Genomics was used to help quickly identify any community cases, to understand how they had arisen, and to determine whether any form of lockdown was needed to maintain elimination.

**Fig. 2:**
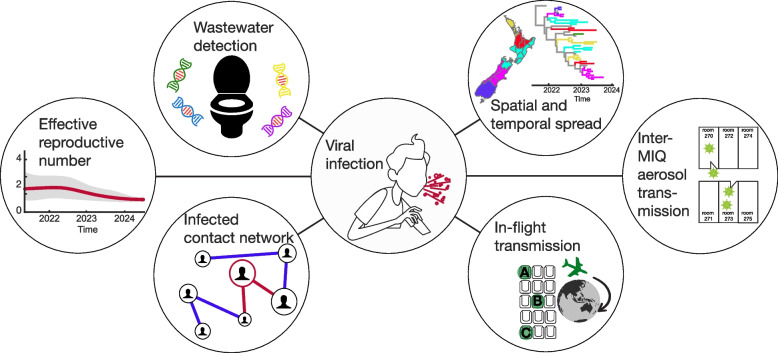
The many modes of genomic surveillance

Here we provide a high-level overview of New Zealand’s genomic journey through this pandemic and discuss the lessons learned for how we should continue to respond to COVID-19 and prepare for future pandemics. Our commentary is aimed at health professionals and decision-makers who might not be familiar with genetic technologies, how they can be used, and why this is an area with great potential to assist in disease detection and tracking.

## Setup and the need for speed

The first SARS-CoV-2 genome was released in January 2020 [5] and publicised on Twitter; it was a prelude to the role rapid data dissemination was to play in the pandemic. Like many other countries, New Zealand scrambled to order primers and reagents to test for, and sequence, the virus [6]. Early on, the decision was made to focus on rapid genomic workflows [7] to respond to the rapid spread of the virus and clarify the mode and tempo of transmission. It would be misleading to say the need and utility for rapid genome sequencing were universally recognised here in New Zealand as the pandemic started. The refrain ‘we already have a genome, why do we need more’ was (too) frequently encountered. We looked with envy at the capacity and resources of the COVID-19 Genomics UK (COG-UK) consortium [8]. However, aided by some emergency, competitive research funding and the benefit of low case numbers, New Zealand embarked on its inaugural real-time genomic journey. Early on, the aim was simple: sequence every case possible and bring real-time genomics to the forefront of pandemic decision-making and contact tracing. Initially, genomics involved integrating results with epidemiological data in a nationally consistent manner. At the same time, pilot funding was provided to explore the possibility that wastewater surveillance, a tool first used over 80 years ago for polio [9] and applied more recently to monitor antimicrobial resistance [10], might assist in pandemic management. For better or worse New Zealand’s capacity in genomics was built steadily, having to justify its utility and value for money.

## Person-to-person genomic links

For the first 18 months of the pandemic, New Zealand’s genomics efforts focused on tracking person-to-person transmission chains, assisting hundreds of cases where transmission routes were unknown or uncertain. Most of these cases only exist in the operational memory of those involved and interim reports to authorities conducting contact tracing.

By sequencing most (typically > 90% (Fig. 1)) of the country’s COVID-19 cases the modes of transmission could be established. For example, genomic surveillance from aeroplane passengers demonstrated the very real risks of in-flight transmission [11]. Testing identified seven people in MIQ who had arrived in New Zealand on 29 September 2020 from Dubai, United Arab Emirates. Despite originating from five different countries before a layover in Dubai, and five of the seven passengers having a negative pre-departure test result, the SARS-CoV-2 genomes obtained from the passengers were genetically identical, except for a single mutation in 1 sample [11].

Similarly, cases within MIQ facilities suggested airborne rather than fomite (surface) transmission [12, 13]. For example, solo traveller A and a 5-person travel group, BCDEF, travelled to New Zealand (July 2021) on different flights from different countries, arrived on different dates, and were housed in different MIQ hotels [13]. While asymptomatic, person A tested positive at their routine day 1 test and was transferred to room 277 of a managed isolation facility. Subsequently, individuals (B, C, D and E) accommodated in adjoining rooms (276 and 278) on the opposite side of the corridor tested positive and were linked by genomics to traveller A. Closed-circuit television (CCTV) showed room doors were opened in short succession of each other, making airborne transmission the only plausible explanation [13].

The critical feature of using genomics was the ability to definitively link (or rule out) transmission even though cases may have been in close proximity. This data, in turn, assisted in developing protection protocols and a better understanding of transmission risk factors. With so few infections, and few transmission chains, New Zealand became an excellent place to study outbreaks of COVID-19 [14]. Integrating genomics with detailed epidemiological data allowed us to explore how, when, and where the virus was being transmitted and how many times it evaded border protections.

## Genomics under elimination

The elimination phase of New Zealand’s COVID-19 response involved tracking every case possible with the aim of stopping (or slowing) transmission chains (Fig. 1). At its core, the strategy was designed to buy time for the development and roll-out of COVID-19 vaccines and therapeutics. After the initial country-wide lockdown in 2020, and with international arrivals only coming in via MIQ [2], the aim was to quickly identify any community cases and understand how they had arisen. That meant that through 2020 and 2021, traditional contact tracing methods were ramped up, and were increasingly complemented by genomics [14].

When cases arose, the race was on to understand if there had been single or multiple introductions. This is where genomics shone. With each new infection, the virus slowly accumulates point mutations. These genetic signposts provided the ‘clues’ to link cases to clusters, and clusters to the border. As the history of individual cases was investigated, analysis of diagnostic mutations enabled the exclusion of some transmission routes and the likelihood of others to be established. Genomic data also highlighted the risks of super-spreader events and even enabled estimates of how long a given transmission chain might have been active in the community before it was detected [15, 16]. This informed government decision-making about when a lockdown was necessary to maintain elimination.

New Zealand’s contact tracing system (EpiSurv) was not well set up to accept genomic data or intelligence. Instead, a set of ad-hoc and time-intensive ‘work arounds’ were devised to recover the most from genetic data. The relatively small number of cases enabled this to occur but in the future systems that better integrate epidemiological and genomic data will need to be developed. This is something that has long been called for [17–20], and the work of Forde and colleagues using genomic surveillance to rapidly identify the transmission of multi-drug resistant bacteria within healthcare settings in Queensland, Australia, provides an excellent exemplar [21].

To complement genomics the Institute of Environmental Science and Research (ESR) was also developing its capability in wastewater-based surveillance to detect SARS-CoV-2 RNA being shed by infected and recovered COVID-19 cases. Unlike many jurisdictions around the globe, the low number of cases under elimination meant that, except for wastewater from quarantine facilities, results were largely negative across the country. With close monitoring of cases in isolation it was possible to develop a feel for the sensitivity of the method. Initially, the primary aim was therefore to develop ultrasensitive detection of SARS-CoV-2 RNA [22]. The impact of wastewater-based surveillance, including New Zealand’s examples of ‘early warning’, is now reflected in the World Health Organisation’s 2022 guidance on environmental surveillance for COVID-19 [23]. Following the arrival of Delta (late 2021) wastewater became less about presence/absence and more about the levels of viral RNA in the wastewater and variant detection.

## Genomics under suppression

In late 2021, during the long tail of a Delta outbreak, New Zealand abandoned elimination in favour of suppression due to high vaccination coverage and the inability to eliminate Delta, despite stringent public health settings [16]. It immediately became impossible to sequence a high proportion of cases (Fig. 2). The questions were no longer about transmission chains, but rather now centred around variants (including their disease severity), persistent infections, and the dynamics of viral spread. Under suppression, New Zealand focused its genomics capacity on four key areas of genomic surveillance: (i) border cases to detect the arrival of new variants; (ii) hospitals, to detect variants possibly linked to increased disease severity; (iii) a random proportion of community samples; and (iv) targeted groups (for example, persistent infections or geographic regions associated with the detection of a new variant). Changes in the percentage of border and community cases sequenced through the different phases of the pandemic are shown in Fig. 2. Through 2022, ESR sequenced on average 32% of hospital cases (admitted for any reason). The ad-hoc nature of targeted group surveillance (for example, persistent infections) means it is not possible to report statistics. Likewise, as the system pivoted to suppression, wastewater-based surveillance now focused on quantifying viral RNA and identifying what variants were circulating.

One of the first challenges under the suppression strategy was dealing with the arrival of Omicron. With a well-vaccinated population in late 2022 [3], New Zealand had nearly eliminated Delta; the seven-day rolling average was under 60 confirmed cases per day [3]. The detection of Omicron by the South African surveillance system [24] changed everything. Our genomic surveillance first detected Omicron at the border in early December 2021, with community cases following in late January 2022. Genomic surveillance demonstrated that BA.1 was the first Omicron variant to arrive in New Zealand [25]. BA.2 followed soon after and its transmission advantage saw it rapidly displace BA.1. Genomics demonstrated that in the first half of 2022, approximately 85% of all New Zealand cases were BA.2 [25]. Increasingly, as a conveyor belt of variants emerged, the country sought to use its genomic surveillance to also plot the infection history which was set to become a determinant in the susceptibility or resilience to subsequent waves.

By March 2022, the MIQ system was dismantled, and New Zealand opened to citizens, permanent residents and work visa holders without the need to self-isolate until testing positive [4]. Pre-departure tests were dropped by mid-June 2022, coinciding with the arrival of subvariants BA.4, BA.5, BA.2.12.1 and BA.2.75, first at the border, then in the community [25].

The waves of Omicron (sub)variants highlight the importance of genomics in understanding re-infections and persistent infections. At the time of writing (late 2022), we were seeking to understand if people previously infected with BA.2 (the bulk of New Zealand’s Omicron cases in 2022) were susceptible/resilient to reinfections with BA.5, BA.2.75, BA.4.6 or BQ.1. Without a genomic lens, it would not be possible to even start addressing these questions. Likewise, genomics provides the ability to understand how the virus is evolving within persistently infected hosts [26–29]. Our genomic surveillance system currently prioritises such cases for genome sequencing and detected some patients with new combinations of spike protein mutations that we watched carefully.

In 2022 as border restrictions were progressively scaled back across New Zealand, we were able to observe a change in state from that in which a single lineage from a single introduction spread across the country, to the situation we have today where there are regular border ‘jumps’. In mid-2022 genomic surveillance estimated that there was ~ 1 border ‘jump’ per 5000 passenger arrivals, or about 2 per day [25]. These metrics provide modellers with estimates of the speed at which variants arrive, spread, and their fitness advantage.

On the wastewater front, ESR set up an interactive dashboard that set out to (with permission) geolocate people and show them the wastewater trends in their local catchment [30]. The wastewater system provides quantitative trends for ~ 75% of New Zealanders across the country updating trends on a weekly basis at over 100 sites. Increasingly the under-reporting of COVID-19 meant that wastewater surveillance (with appropriate modelling) provided much-needed estimates of disease prevalence and trajectories. The involvement of modellers further enhanced forecasting and was able to interweave wastewater and epidemiological data to estimate metrics such as effective reproductive number (Re) (Leighton Watson (University of Canterbury, New Zealand), personal communication).

Variant analysis from wastewater was also put in place with a focus on identifying genetic variation within the SARS-CoV-2 spike region (primarily the receptor binding domain). There are multiple global strategies for the detection of variants in wastewater; in early 2022, ESR in collaboration with an eDNA specialist (Wilderlab, New Zealand) adopted a ‘short amplicon’ approach that prioritised (i) sensitivity (as wastewater RNA is degraded) and, (ii) quantitative signal so that the proportion of each variant could be explored. This approach was able to track the BA.2 to BA.5 transition with excellent precision relative to genomic surveillance of clinical cases (the R2 correlation was > 0.95, ESR, unpublished data). Tracking the variants in wastewater became much more difficult when the number of Omicron variants increased rapidly at the end of 2022. However, our experience was that there was excellent complementarity between genotypes derived from both whole genome sequencing and wastewater variant analysis and the benefits of looking at these data in tandem.

Like rapid genomics, the establishment of a wastewater-based surveillance system is yet another capability that New Zealand has gained through this pandemic. The challenges of establishing and maintaining such a system have been discussed elsewhere [31–33] and include resourcing [34] as well as developing and maintaining relationships with the entities responsible for wastewater infrastructure, especially if these change ownership over time. During COVID-19, New Zealand was able to leverage the urgency and seriousness of the pandemic alongside existing relationships developed for the surveillance of elicit substances from wastewater [35, 36]. As is being done elsewhere [37–39], in New Zealand the archive of wastewater samples amassed during the pandemic is now being interrogated for other pathogens such as the viruses responsible for polio and monkeypox. Storage of such samples could represent an invaluable archive (and baseline) for the study of human health and disease in the years ahead.

One important tool that New Zealand has planned, but is yet to implement, is a genomically-integrated prevalence survey. In the UK, for example, the Office for National Statistics (ONS) Coronavirus (COVID-19) Infection Survey [40] has provided regular snapshots of how many people have COVID-19 and which variants they are infected with, as well as providing important information on real-world vaccine efficacy. In addition to data from wastewater and patient samples, in New Zealand, we are reliant on an opt-in weekly online symptom survey called FluTracking which has been monitoring influenza-like symptoms in Australia since 2006 and in New Zealand since 2018 [41]. The benefits of prevalence surveys will assist in many aspects of pandemic management including estimates of case under-ascertainment and calibration of wastewater quantitation to infection rates.

## The future of genomic surveillance

With the benefit, insights, and hard work of many around the globe, New Zealand has forged a small yet agile genetics/genomic surveillance capability. Arguably, more so than in other countries, we have used many modes of genomic surveillance: from tracking hundreds of transmission chains in the community, aeroplanes and within quarantine facilities—to assist in contact tracing—through to monitoring the mode and tempo of spread across cities and sewer sheds. Deliberately, New Zealand has chosen to focus on turnaround time to assist in real-time decision-making. In 2020 and 2021, most samples were turned around in a few days. In 2022, Aotearoa New Zealand remained in the “Very Frequent/Very High” quadrant in a comparison of countries assessed on the basis of their frequency of sampling and the overall percentage of cases sequenced [42], with less than 1% of samples having a turn-around time of more than 14 days (ESR, unpublished data). We advocate that workflows designed with speed in mind are more conducive to the journey towards point-of-need diagnostics and agile decision-making. This pandemic has demonstrated that real-time genomics is a reality; it has prompted questions surrounding how best to adapt our COVID-19 learnings to monitor other infectious diseases as well as the looming crisis of antimicrobial resistance.

We also advocate that our genomic surveillance needs to extend well beyond the scope of patient swabs and wastewater. Detection of disease in the air (for example, via ventilation systems in buildings or aeroplanes), surfaces (for example, sampling public stairwells or escalators) and even within our animal populations [43] is needed to build a genomic surveillance system with as few gaps as possible.

New Zealand has benefitted from the methods and data generated on the international stage as we have sought to understand variants and understand the geographical origins of viral lineage as they arrived. In an effort to share data, ESR uploaded genomic data to the GSAID database once or twice a week and often shared data with Australia in real-time to manage transmission risks.

Like many other jurisdictions, the monitoring of COVID-19 (and for other pathogens) at the human-wildlife interface is an area where New Zealand is lacking. While such surveillance has its challenges [44, 45], there are numerous excellent exemplars [46, 47]. Global initiatives such as PREZODE (stood up in response to COVID-19) are gaining traction yet still seem underfunded relative to the risk despite the best efforts of OneHealth initiatives. Just one example of why this lack of monitoring in New Zealand concerns us, are the reports of sustained and accelerated evolution of SARS-CoV-2 in deer, and of deer-to-human transmission [21, 22]. New Zealand has a significant population of wild and farmed deer.

Finally, innovation in how, when, and where we deploy our genomic surveillance toolkit will determine how ready and rapidly we are able to respond to the next infectious disease challenge. One key learning is that there is no single ‘mode’ of genetic surveillance, it must remain agile and ready to respond at short notice. Hand-in-hand with this agility is the need to make the explanation of genetic results accessible to a wide range of decision-makers and health professionals. Within the New Zealand health system, the integration of genomic technologies has not been seamless. In the future, we see that more accessible interfaces with data are still needed, as is the ongoing training of people who need to interweave genomic data into their decision-making.

## Conclusions

While New Zealand, like many other countries, has realised the enormous benefit of using genomics and wastewater surveillance during a public health crisis, we are keen to see the learnings and knowledge gained as an opportunity ‘springboard’. Prior to COVID-19, terms like ‘genes’ and ‘genomics’ in New Zealand likely conjured images of genetically modified organisms from polarising debates of two decades earlier. The use of mRNA vaccines and the well-publicised setup of real-time genomic tools for pandemic tracking and decision-making have brought the technology into the public eye. Now, phrases like PCR, CT values, and variants have become common, even used by then Prime Minister Jacinda Ardern during televised press briefings. We envisage a future where New Zealanders are better informed about the benefits (and risks) of embracing genetic technologies, how RNA-based therapeutics are game-changers, and how a health system where genomic data is integrated in an accessible way should be our preferred future pathway.

## Data Availability

Not applicable.

## Abbreviations

CCTV: Closed-circuit television
COG-UK: COVID-19 Genomics UK
COVID-19: Coronavirus disease 2019
CT: Cycle threshold
ESR: Institute of Environmental Science and Research
GISAID: Global Initiative on Sharing Avian Influenza Data
MIQ: Managed Isolation and Quarantine
mRNA: Messenger ribonucleic acid
ONS: Office for National Statistics
PCR: Polymerase chain reaction
RNA: Ribonucleic acid
